# A (mis)guidance of disabled youth: Post-secondary schooling transition experiences in South Africa

**DOI:** 10.4102/ajod.v12i0.1293

**Published:** 2023-11-14

**Authors:** Armand Bam, Samantha Kriger, Zelda Cottle

**Affiliations:** 1 Stellenbosch Business School, Faculty of Economics and Management Science, University of Stellenbosch, Cape Town, South Africa; 2 Department of General Education and Training (GET), Faculty of Education, Cape Peninsula University of Technology, Cape Town, South Africa

**Keywords:** disabled youth, employment, inclusion, transition, career guidance

## Abstract

**Background:**

Globally, there is a disparity that exists between equal employment opportunities for people with disabilities post-schooling. While South Africa has aimed at the inclusion of people with disabilities, there has not been sufficient evidence of a successful transition from school to work environments.

**Objectives:**

This study documents the experiences and barriers that influence the preparation of high school students with disabilities for post-secondary education and work opportunities.

**Method:**

A qualitative research methodology employing multiple case study design was used where semi-structured in-depth interviews were conducted with youth between the ages of 18 years and 36 years who were currently employed. The participants were identified through purposeful sampling. Data were analysed by thematic analysis.

**Results:**

The findings indicate two overarching themes indicating that the career choices of participants, firstly, were significantly influenced by teacher and guidance counsellor expectations and, secondly, experiences of discouragement where personal agency and autonomy were limited.

**Conclusion:**

This study illuminates the need to enhance the decisions around careers for people with disabilities which should debunk the expectations of society.

**Contribution:**

This study will make teachers, mentors and counsellors more aware of their contribution, influence and support to youth with disabilities as they transition into the workplace.

## Introduction

Despite developed inclusion legislation, transition policies and financial resources, a failure to promote entry to further education, economic opportunities and independent living for people with disabilities persists in South Africa (SA) (Bam & Ronnie 2020). Although more people with disabilities are able to gain access to educational opportunities, high school graduation rates in general for people with disabilities continue to lag behind that of non-disabled peers (Francis et al. 2018; Kirby 2017; Kloos, Nacik & Ward 2022). Moreover, high dropout rates remain a leading contributor to the deepening social, environmental and economic exclusion that people with disabilities experience (Lindsay et al. 2019). In the global South, dropout rates within the education system are particularly high during transition phases (Hanushek, Lavy & Kohtaro 2008), and because of the coronavirus disease 2019 (COVID-19), this has been exacerbated (Inglis 2023). This in effect has contributed to higher levels of unemployment and lower wages payable for youth with disabilities (Dhakal, Connell & Burgess 2018). For youth aged 15 years–24 years, unemployment sits at 60.7%, and for youth aged between 25 years and 34 years, it hovers at 39.8% (Statistics South Africa 2023). Transitioning from high school for youth with disabilities is therefore challenging making the impact of transition programmes crucial to overcome the societal, environmental, intrinsic, tertiary education and employment barriers.

Globally, transition-specific policies and programmes aim for positive outcomes, where there is a meeting of scholar goals and post-school successes (Mazzotti et al. 2021). Career guidance is part of a life-long journey and a process for the specific deliverance of ‘support in relation to development, choice and placement in educational options and occupations or work roles’ (Van Esbroek 2019:36). Career guidance forms an integral part of the socio-emotional development of youth with disabilities affecting their employment decisions and socio-economic inclusion in society (Lipka, Forkosh Baruch & Meer 2019). Other significant sociological impacts include the effect on confirming certain roles in adulthood, reduced independence and the lack of firming personal identities (Hirano et al. 2018). Furthermore, career planning occurs mainly in high school (Kelechi & Ihuoma 2011) where teachers or counsellors help with identifying career paths (Dislere & Vronska 2020), aligning interests and abilities (Milosheva et al. 2021), providing relevant information on careers (Wong, Yuen & Chen 2021) and determining goals and possible plans to achieve these goals (Ogenyi, Ajibola & Ojochogu 2020). The failures in transitioning from high school are typically ascribed to the uncoordinated nature of these services, poorly designed programmes, lack of opportunities and the disjointed career service provision throughout the life of people with disabilities (Hall & Parker 2010; Kline & Kurz 2014; Santos, Kupczynski & Mundy 2019; Shogren & Wittenburg 2020).

While it is widely accepted that research on transitioning frameworks, models and theories has evolved over time (Carter et al. 2009; Elder 1994; O’Brien & O’Brien 2000; Ryan & Deci 2000), a lack of focussed studies delivering evidence for improving practice remains (Lindsay et al. 2019). One specific crisis area requiring deeper exploration is that of career guidance and the post-secondary schooling impact for people with disabilities (Trainor et al. 2020; Wehmeyer et al. 2019). Furthermore, research on transitioning experiences into employment from the perspective of people with disabilities remains scant (Shogren & Wittenburg 2020), particularly views of people with disabilities from the Global South (Lourens 2020). South Africa as a signatory to the full protocol of the United Nations Convention on the Rights of Persons with Disabilities (United Nations 2006) and its envious inclusionary policies and legislation still manages to see dire results and outcomes for youth with disabilities (UNICEF 2021). South Africa is politically and economically a force to reckon with within sub-Saharan Africa but is still beset with inequalities in all spheres of society. Its political history is tainted with the effects of apartheid having cemented structural and social divisions based on a contrived racial classification system. The remnants of these effects can be seen in the education system and more so in special education schooling where separate schools were in place for white children and children of colour (Walton & Rusznyak 2014). The apartheid policy on special schools ensured that ‘schools that accommodated white disabled learners were extremely well-resourced, whilst the few schools for black disabled learners were systematically under-resourced’ (Department of Education 2001b:9). Few empirical studies have examined the views of people with disabilities regarding their transitioning experiences and the impact on career choices and trajectory once in employment (McLaughlin 2023; Then & Pohlmann-Rother 2023). This qualitative multiple case study therefore sought to address a specific gap in our understanding of the influence of career guidance on post-schooling career trajectory for youth with disabilities in South Africa.

## South Africa’s legislative framework

**FIGURE 1:**
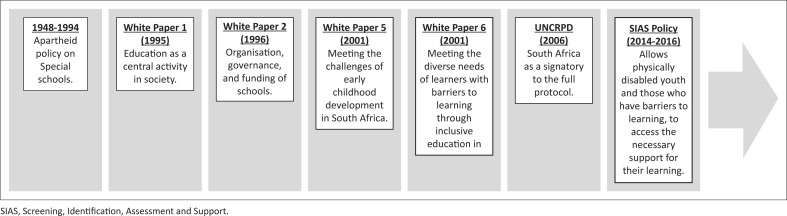
Overview of South Africa’s inclusive education legislative development.

The Constitution of South Africa (Republic of South Africa 1996) provides an all-embracing account of the rights attributable to citizens with disabilities including unfettered access to education. Furthermore, South Africa’s White Paper on an Integrated National Disability Strategy requires that ‘legislation should comply with and give substance to Constitutional requirements’ (The Office of Deputy President 1997:7). In support of this edict, the White Paper 6 on Special Needs Education exposes the national and systemic challenges experienced with building an inclusive education and training system in South Africa (DoE 2001). Within a policy context, White Paper 1 detailing education as a central activity in society (Department of Education 1995); White Paper 2 detailing the organisation, governance and funding of schools (Department of Education 1996); and White Paper 5 detailing on meeting the challenges of early childhood development in South Africa (Department of Education 2001a) provide a specific educational focus that impacts on school going children with disabilities. With a sound legislative framework, it therefore remains a travesty that people with disabilities face persistent challenges in accessing quality education and skills development to support their transitioning through the education system (see Figure 1).

**FIGURE 2:**
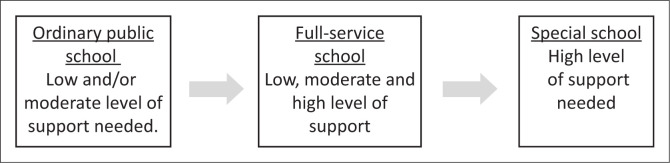
Overview of school types in South Africa.

In South Africa, learners with disabilities can complete their schooling in three categories of schools, namely, public ordinary schools, ordinary full-service or inclusive schools and special schools with two variations (see Figure 2). A ‘placement’ at a specific category of school is inherently linked to the perceived level of support required for inclusion in the specific environment (Agran et al. 2020; Elbaum 2002; Hocutt 1996; Shah 2007). Public ordinary schools or mainstream schools are most welcoming of students who require the lowest levels of support without formal assessment and provide a varied curriculum. In contrast, children attending an ordinary full-service school must undergo a multidimensional assessment evaluating the environmental barriers experienced where teaching and learning are focussed on strengths and competencies (Walton 2011). These ordinary full-service or inclusive schools are defined as ‘schools that will be equipped and supported to provide for the full range of learning needs among all our learners’ (Department of Education 2001b:22). Full-service schools offer flexibility in teaching and learning together with educational support to learners and educators (Ayaya, Makoelle & Van der Merwe 2020) and embody the principle of diversity and fostering maximum participation. While inclusion through full-service schools is encouraged, South Africa still experiences a shortage of suitably qualified teachers with special education training (Kempen & Steyn 2016; Ladbrook 2009; McKenzie et al. 2023). Significantly, specialised teacher support is linked to improved learning outcomes, positive attitudes to learning and better career development outcomes (Wong et al. 2021), and within a South African policy framework, this central role of the teacher is acknowledged (Department of Education 2001b).

Two forms of special schooling opportunities exist. Firstly, the traditional ‘exclusionary’ special school is associated with a specific impairment (blind, deaf, physical disability, etc.) for children with high-intensive educational support needs on a permanent or part-time basis. These schools have recently been encouraged to share their expertise and provide support services to ordinary and full-service schools. Secondly, certain neighbourhood schools have been designated as resource centres that are integrated into district-based support teams (Department of Education 2001b). These schools are ‘transformed to accommodate learners who have high intensity support needs and designed to assist learners through integrating various teaching methods based on the individual student’s needs’ (Department of Education 2001b:20). While the special schooling system aims to support children with disabilities questions remain about its suitability to facilitate access to tertiary education and job opportunities later in life (Mitra 2018).

## Conceptual framework

Students with disabilities tend to have lower levels of post-school success than their non-disabled peers (Mazzotti et al. 2021). While there do not appear to be standardised approaches to transitioning, there is a common belief that youth with disabilities are better able to meet their goals when exposed to an appropriate mix of support, services, instruction and opportunities (Carter et al. 2009). Transition into adulthood represents a critical step imbued with confusion and difficulties when not planned appropriately (Dell’Armo & Tassé 2019). Bronfenbrenner’s ecological systems theory shows how the outcomes of transitions are influenced by the interactions of various key elements including educators, family, professionals, policies and societal expectations’ impact on the outcomes (Lindsay et al. 2018). Thus, transition planning at high school has a lifelong impact (Nuske et al. 2019) and is a significant indicator and determinant of success for tertiary education and desired careers (Brooke, Revell & Wehman 2003). Transitioning is complex, and through the influence and interactions of multiple systems and their interactions that include the microsystem (family, school), mesosystem (connection between family and school), exosystem (policies and services), macrosystem (societal beliefs and values) and chronosystem (historical context), a holistic approach can be adopted to support students with disabilities during the transition process.

Beyond acknowledging the multiple systems influencing people with disabilities transitioning, personal agency, a concept central to Albert Badura’s social cognitive theory, plays a critical role too. Bandura (1989, 1997) emphasised the influence of self-efficacy beliefs and the capacity to exercise control of personal actions and choices. Moreover, Shogren and Wittenburg (2020) found that customised and narrowly focussed programmes in line with Bandura’s principles reinforce the need for early intervention directed by the youth’s needs. Through promoting autonomy and self-determination, it becomes evident that offering choice, motivation and self-determined outcomes can promote wellness and happiness (Dunn & Brody 2008). While studies on decision-making and expectations in the context of people with disabilities are widely examined, the tertiary education and employment outcomes for youth with disabilities still remain bleak (Broberg 2011; O’Brien & O’Brien 2000; Kirby, Dell’armo & Persch 2019; Russell 2003). Bandura’s theory acknowledges the impact of environmental influences, such as societal norms and expectations, on career outcomes. Therefore, it is critical to address these environmental factors to enhance the transition experiences of youth with disabilities. Considering the layered and textured experience of transitioning, successful outcomes call on students to be placed at the centre of all processes (Nuske et al. 2019). Through encouraging youth with disabilities to evaluate their own decisions as well as others, self-determination and autonomy are promoted – key elements emphasised by Bandura – for ensuring the maximum participation in post-secondary education and employment (Deniz 2023; Gobec, Rillotta & Raghavendra 2022). Integrating these concepts in the transitional processes can empower youth with disabilities to overcome challenges and experience more favourable career outcomes.

Guidance and career counsellors are critical in developing the necessary understanding for better career choices. Oliver’s (1990, 2013) social model of disability recognises that disability results from more than an individuals’ impairment and is also shaped by attitudes, systems and environments. This framework promotes equal opportunities and support for people with disabilities in making informed decisions about their careers and in so doing laying emphasis on the critical role guidance and career counsellors play in schools. Career prospects for disabled youth are largely influenced by the school they attend and the quality of the support and resources available (Klang et al. 2020). In line with Oliver’s views, the need to ensure environments that are accessible physically, free from discrimination and offer inclusive support systems is necessary for the successful transitioning into careers or further education (Hirano et al. 2018; Lindstrom, Doren & Miesch 2011; Stuntzner 2014). An educational experience free of such barriers is important in ensuring individuals can participate fully in society and access diverse career options.

In the South African education system, there continue to be blurred lines regarding disability. It is for this reason the Policy on Screening, Identification, Assessment and Support (SIAS) was documented in 2014 but implemented only from 2016 (Department of Basic Education 2014). From a social model perspective, this policy represents a move towards recognising the need to provide support to those students experiencing barriers to learning. Practical implementation remains a challenge, and the persistent gap between policy intent and provided support is detrimental to progress. Furthermore, from the social model perspective, the development of inclusive policies and the effective implementation to ensure equal access and support must be coordinated (Van Niekerk, Maguvhe & Magano 2022). Teachers who teach special needs students need to have the pedagogical knowledge as well as expertise in teaching learners with special needs as this is vital for the learning experiences of such children (Karisa, McKenzie & De Villiers 2020). ‘This implies that high quality teacher education and development programmes are required for teachers in special schools to ensure optimum learning experiences for learners in special schools’ (Gorman & Drudy 2011).

The conceptual framework considers the multifaceted nature of this critical phase in people with disabilities lives. The framework combines elements of ecological systems theory, social cognitive theory and the social model of disability and offers a holistic perspective considering the complex interplay of various factors shaping the transition experiences of youth with disabilities. The ecological systems theory, as proposed by Bronfenbrenner, underscores the importance of examining the interactions between educators, family, professionals, policies and societal expectations in influencing outcomes during the transition process (Lindsay et al. 2018). By recognising the influence of multiple systems and their interactions, we can adopt a more holistic approach for effective support. Bandura’s (1997) social cognitive theory highlights the need for tailored interventions empowering youth with disabilities to make choices and foster self-determined outcomes that can significantly enhance their overall well-being and happiness. Moreover, the social model of disability emphasises the role of attitudes, systems and environments in shaping disability. This perspective aligns with the idea that career prospects for disabled youth are heavily influenced by the quality of support and resources available in educational settings.

Drawing from the ecological systems theory, social cognitive theory and the social model of disability, therefore, offers a nuanced understanding of the transition experiences recognising the complexity of this process and the various factors at play, from interpersonal interactions to societal structures. In the context of South Africa, where the implementation of inclusive policies remains a challenge, this framework can shed light on the gap between policy intent and practical support. It underscores the need for coordinated efforts to ensure equal access and support for students with disabilities. Additionally, it highlights the importance of high-quality teacher education and development programmes to create optimal learning experiences for these students. Through considering this holistic perspective, our study is aimed at empowering youth, improving their outcomes and creating a more inclusive society for all through addressing how people with disabilities experience career development practices.

## Research methods and design

The case study design was employed to permit the researchers to gain in-depth knowledge about a specific real-world problem; in this instance, how youth with disabilities experience career development practices. Case study is viewed as a suitable research design when the proposed research focuses on a contemporary phenomenon, which the researcher has no control over; the research is mainly exploratory; and it addresses the ‘how’ and ‘why’ questions (Darke, Shanks & Broadbent 1998; Yin 1994). For this research, an exploratory multiple case study approach was deployed to explore the transition experiences of employed youth with disabilities and how career guidance impacted their career trajectory. A case study is an empirical enquiry that explores a modern phenomenon within its real-life context, especially when the boundaries between phenomenon and milieu are not apparent (Yin 1994).

The selection of cases focused on employed youth with disabilities who had attended special schools in South Africa (Etikan 2017). It is widely established that the selection of case studies need not be a static activity; however, the process needs to be justified, fully detailed and later stated to the case study audience in order to provide the context for determining the sample (Morse 2020; Yin 1994).

The inclusion criteria for participants in this study (Table 1) were:

- Youth (18–36) with disabilities who can converse in English or Afrikaans
- Both male and female participants
- Were employed for at least 1 year at the time of the study.

**TABLE 1:** Case profiles.

Pseudonym	Gender	Age	Relationship	Highest qualification	Duration of employment	Type of impairment	Onset	Cause
Justin	Male	33	Single	Degree	3 years	Quadriplegic	Late	Fractured C4-vertebrae
Kate	Female	29	Married	Master’s	2 years	Cerebral palsy	Early	Spastic Diplegia
Lauren	Female	28	Single	Degree	3 years	Visually impaired	Early	Unknown
Mthokozisi	Male	34	Married	Matric	7 years	Paraplegic	Late	Fractured C4-vertebrae
Muneeb	Male	29	Single	Degree	7 years	Spastic diplegia	Early	Loss of oxygen at birth
Renier	Male	31	Single	Master’s, PhD (in progress)	4 years	Visually impaired	Early	Unknown
Tania	Female	25	Single	Degree	5 years	Osteogenesis imperfecta	Early	Brittle bone
Wouter	Male	32	Single	Master’s	5 years	Visually impaired	Early	Unknown

Data were collected by way of eight semi-structured in-depth interviews to explore and understand the perceptions of disabled youth on career development practices to the point of saturation. Where time was limited, a follow-up interview was conducted for further clarification. Data were collected over 3 weeks and advanced to the primary analysis when the data were still fresh. Each interview was 45 min to 1 h long.

### Data analysis

The researchers applied the six-step framework suggested by Braun and Clark (2006) in the thematic analysis of data. The approach accommodated the ability of all researchers to engage with the analysis in a pragmatic manner (Aronson 1994). One researcher transcribed the raw files verbatim after each interview session ensuring that the first step of data familiarisation was achieved. The data corpus therefore consisted of all transcribed interviews (Merriam 2009). The transcripts were shared with co-researchers once completed. The primary investigator initiated the second step of the process conducting the first level of coding and translating the raw data into meaningful groupings (Evans & Lewis 2018). The process involved a sequence of grouping similar data where key concepts were extracted from the participants’ responses. The third step was for the primary investigator to examine the transcribed interview responses in order to identify patterns of similarities and dissimilarities in the perceptions of the participants (Skjott Linneberg & Korsgaard 2019) and then subsequently compare them to each other. In the fourth stage, the primary investigator explored the themes and added codes and furthermore assessed how they interconnected with each other (Braun & Clarke 2006; Neal et al. 2015). During the fifth phase of theme modification, the primary investigator identified areas of interest in relation to the study at hand (Braun & Clarke 2006). The sixth and final phase is the presentation of findings. As a means of triangulation, the researchers met to confirm the codes, categories and themes presented as well as reviewed existing literature to confirm alignment. Furthermore, the trustworthiness of the study was developed through providing participants opportunities to review our transcriptions and maintaining an audit trail of our decisions through thematic analysis.

### Ethical considerations

An application for full ethical approval was made to the university Research Ethics Committee, and ethics consent was received on 04 October 2021. The ethics approval number is SU-23443. The recruitment process commenced after this study was approved by the university Research Ethics Committee under project number: USB-2021-23443. All procedures performed in this study were in accordance with the ethical standards of the institutional committee and with the 1964 Helsinki Declaration and its later amendments or comparable ethical standards. Written informed consent was obtained from all individual participants involved in the study. Confidentiality was maintained throughout the study, and anonymity was addressed through providing participants with pseudonyms.

## Findings

Interviews conclude with a synthesis of findings representing participants’ accounts of the career guidance received and factors that impacted on their career trajectory and transitioning as youth with disabilities having attended special schools. The findings shared are arranged according to two overarching themes and six sub-themes (Table 2). The main themes addressed in this article include firstly, *Tailored expectations,* addressing the discouragement experienced by participants related to teacher expectations and personal agency and autonomy. The second theme, *Mis(Guidance) curriculum,* captures the influence of the advice provided by counsellors, the resistance engendered through negative experiences, the need to enhance career decision-making and the societal expectations on youth with disabilities.

**TABLE 2:** Factors impacting on career choices for youth with disabilities.

Theme	Category	Codes
Tailored expectations	Teacher expectations	Internalised expectations and inclusion
		Uplifting or underestimating potential
	Being seen	Subjective determination
		Active discouragement
		Projecting personal fears
		Reduced autonomy
(Mis)guidance curriculum	Guidance and advice	Suited to disability
		Ambiguous advice
		Entrenching social norms
	Resisting authority	Resisting pressure
		Perseverance
		Expanding sources of career advice
	Enhancing career decision-making	Communal inputs
		Dialogical engagement
		Collective responsibility
	Societal expectations	Perpetuating negative stereotypes
		Normative behavioural outcomes
		Lowering standards

### Theme 1: Tailored expectations

Teacher expectations are critical in the development of children with disabilities educational experiences. Besides family and close friends, school teachers’ expectations were internalised by all participants;

‘But I think, for some of the people who aren’t used to the idea of inclusion, I think that was a bit, yeah, and it was obvious, but I got over it.’ (Tania, female, 25, Osteogenesis imperfecta)

became a reference point of inclusion or othering;

‘And I understand people being skeptical … it is much harder [*being disabled*] … you know … [*they believe*] odds are low that you’ll make it and it’s a numbers game to get to university.’ (Wouter, male, 32, Visually impaired)

and experienced as uplifting or undermining of their potential throughout their school journey.

‘But another aspect is that a lot of times disabled people are not encouraged to study careers like this. Because it’s assumed by the educators that they will struggle, which they will, but that’s part of the learning process. And also, it’s assumed by the educators that a lot of material and, and so forth, are inaccessible, and therefore it’s going to be too much effort.’ (Renier, male, 31, Visually impaired)

All our participants considered the way teachers received them, included them and encouraged them to have impacted on their choice of tertiary studies and career. Justin’s experience arriving as a new scholar captures these tailored expectations.

‘I was a guinea pig for them because I was the first quadriplegic at the school … That school has everything … though I cannot fault them on being accommodating to my needs, the schoolteachers did not expect much from us and I felt they didn’t believe we could excel beyond grade 12.’ (Justin, male, 33, Quadriplegic)

Despite the specialisation and focus on disability at these schools, participants felt that teachers did not ‘see’ them first as scholars but rather focussed on their disabilities. For many participants (6), teachers often made subjective determinations about their ability, actively discouraged participation in certain school activities and projected their personal fears onto scholars. These factors resulted in experiences of reduced scholar autonomy. Participants’ experiences in this way illustrated the attitudinal barriers encountered within special schools. Participants considered teachers and guidance counsellors to be most influential in motivating their career interest. These teachers needed to consider more than just their perception of their ability to perform in a certain career and had to move beyond an approach of finding more ‘suitable activities’ to avoid undermining scholars’ experience of their disability.

The role of teachers in shaping an interest in specific careers and the discouragement experienced became constant reminders for all participants when considering career choices later in their lives. Untested assumptions of scholar abilities together with the discouragement to participate in various activities had further consequences on what schools would resource and provide as part of a more expansive curriculum and learning and teaching experience. Furthermore, these tailored expectations were felt to be limiting with respect to developing an aspirational mindset for children with disabilities.

### Theme 2: (Mis)Guidance curriculum

Participants (8) were reflective of the role guidance counsellors played in shaping their career trajectories. In class guidance sessions participants felt that counsellors coerced them into considering certain careers that were ‘suited’ to their ‘abilities’ and/or disability, were intentionally vague and ambiguous with career advice and relied on entrenched social norms rather than individualised assessments when providing advice. This (mis)guidance as described by participants (8) led to feelings of discouragement and self-doubt. The limitations imposed through these sessions in most cases impacted on the choices participants made with respect to their tertiary education options. Tertiary education choices invariably impacted on the first professional working opportunities participants experienced:

‘The guidance counsellor persuaded me to lean towards easier school subjects. We were made to feel that we had to act as ‘normal’ as possible. Because of the very conservative culture we were also not allowed to question anything. I wanted to do Information Technology, but it wasn’t part of the syllabus, and the school were not willing to hire a teacher to facilitate this subject.’ (Lauren, female, 28, Visually impaired)

Guidance counsellors were viewed as authoritative figures, and most participants (6) were not inclined to challenge their views and advice. While participants felt discouragement, some believed they needed to resist the pressures to accept without questioning, persevere and persuade despite possible retribution and expand their sources of career advice and guidance to promote their personal development. Resistance was viewed as a necessary tool for self-promotion, and being perceived as rebellious was a necessary consequence to achieve the desired career aspirations. Personal motivation, perseverance and exposure to other sources of career advice played a role in fortifying participants’ resistance:

‘I told her I want to do science and she says, why? They said it isn’t useful, who’s going to employ some blind person doing science … And I said, because I’m interested in it. And then she says, but why, it’s not useful? And then I’m like, I’m going to study something useful. It will be useful. But if I don’t have [*science*] I can’t study stuff in technical, in the science direction, and then she says but you can do political science … So then I just told her, listen, write there on your piece of paper, I didn’t want to listen to you. And one day, you can tell me that I told you so when it comes to that.’ (Wouter, male, 32, Visually impaired)

While most participants (6) expressed the discouraging impacts of guidance counselling, there was an indication when guidance counselling could provide a positive experience and enhance career decision-making. Where sessions were conducted and required communal inputs, positive experiences were possible. The value of dialogical sessions introduced a collective responsibility for improving the understanding of careers:

‘High school was a great time to start thinking about my career. The regular group guidance sessions arranged by our guidance counsellor was helpful for me. In particular the time spent with my peers where we collectively had an opportunity to discuss and explore our career aspirations, the experience was almost like a shared responsibility.’ (Tania, female, 25, Osteogenesis imperfecta)

Guidance counselling services in special schools provide the first exposure to potential career choices for people with disabilities. A structured approach to informing scholars of their potential to develop careers based on their personal interests and not disability in safe and unexposed environments was important to participants. These sessions need to be open and free of judgement for scholars to express their understanding or misunderstandings of prospective careers. The frequency of these sessions also played a role in creating a safe space for the exploration of scholar’s ideas and thoughts. Where these environments did not provide this, resistance and perseverance were required.

Participants (8) raised concerns about how the discouragement of teachers and counsellors to do more than required perpetuated a negative perception of people with disabilities; promoted the observance and prioritisation of normative behaviours and outcomes, and reinforced an understanding that children with disabilities only required the achievement of the ‘lowest standard’ to be accepted in society. For participants, these societal perceptions were still experienced and expressed in special schools:

‘Ending up in a wheelchair … they’re not encouraging you to complete school, they tell you to complete school, but don’t pass all your subjects … you just need five subjects to pass out of the six … that puts me off. There’s no sense of, you are gonna have to do this … you are going to better your own life, it’s just okay. So that is how, that’s society, I’m going to tell you the truth, that is how society sees people with disabilities.’ (Justin, male, 33, Quadriplegic)

While teachers played a role in advising of different career choices, the broader school’s leadership and decision-making bodies also had an impact on the expectations and overall educational offering presented. As the demand for specific subjects declined in relation to the discouragement over time, the schools resourcing of the ‘difficult subjects’ also diminished. Participants believed mainstream schools did not experience this. Moreover, participants suggested that the societal expectations of children with disabilities could also be witnessed through the disparities between historically disadvantaged special schools and others. Providing the appropriate subjects that laid a foundation for certain fields of study was more prevalent even in advantaged schools and had a societal impact as fewer people with disabilities would be able to enter ‘specialised fields’.

‘So that’s the problem we have in South Africa, education for disabled people … it’s really not adequate at all, in an ideal world, this is something that should start from, ground phase. We must be honest, even schools, like X School [*private and government funded school*] they’ve already phased out physical sciences, which means that anyone who goes to X School is not able to study BSc at the university.’

Participants (8) recognised the importance of subject choices in furthering their tertiary education and employment ambitions. While in most cases participants succumbed to the pressures, resistance also played a role in determining the educational outcomes for scholars. While the school teachers and counsellors advised against taking certain subjects, those who rebelled considered the struggle to be worth the effort:

‘Even my friend and I at school, we were advised against taking mathematics and physical sciences, because of the assumption that it is difficult, and we would experience, and it was difficult, but it was worth it.’ (Renier, male, 31, Visually impaired)

There was a tacit recognition by participants that not all special schools provided the same opportunities for scholars, especially children with disabilities from disadvantaged backgrounds who faced greater challenges. Model C schools were known to rely on governing body funding as well as government funding allowing for the opportunity to present a broader curriculum than advised by the state. Renier explained:

‘I would assume due to the people I’ve talked to you, about 90% of blind people are from very rural backgrounds, where they are just not exposed to, careers like this [in] the schools for the blind, and the education for the blind in South Africa. Unless you go to some place that, that, you know, that’s traditionally a Model C school.’ (Renier, male, 31, Visually impaired)

## Discussion

School career guidance counselling plays a crucial role in the decision-making and career trajectories of youth with disabilities. The findings of this study are consistent with research that suggests administrators, teachers and school governing bodies are instrumental in determining the quality of education, environment and experiences of scholars with disabilities (Esposito, Tang & Kulkarni 2019). A further affirmation of the need to consider an ecological perspective when planning transitions was provided. The participants demonstrate that this influence has longer-lasting consequences affecting their social and economic inclusion in society. The career choices made by participants are influenced by teacher and guidance counsellor expectations that at times reinforce a medicalised view of disability, that is, teachers were inclined to believe that a ‘problem’ exists within a person. This study reveals that teachers found it difficult to envision other positive outcomes for scholars as their unconscious biases prevailed, reinforcing the importance of addressing attitudes influencing transitioning in special schools (Oliver 1990). A fallacy enforced disadvantage resulted in most cases akin to what Rosenthal and Jacobson (1968) described as the ‘self-fulfilling prophesy’. Furthermore, we reveal how the prediction of another’s outcomes is arrived at through actions that unintentionally and indirectly influence the behaviours of others, which leads to entrenching disadvantage that youth with disabilities experience. Teachers who believed that key subjects like mathematics and science were undoable discouraged student participation, impacting demand and leading school governing bodies to defund or eliminate such positions. In our study, participants explain they constantly reflect on what they were told they could not do, which has had an effect on their achievement outcomes (Becker & Luthar 2002). Through this study, we show a darker side to counselling that presents itself based on the accounts of our participants having attended special schools. The (mis)guidance of scholars shows the destructive role guidance counselling can have, particularly for youth with disabilities that are already minoritised and care is lacking. The role of career guidance has previously been identified as either ‘preventative’ or ‘re-integrative’ (Watts 2001). The preventative orientation typically occurs while scholars have access to education, and the re-integrative orientation occurs when outside of such structured educational opportunities. Both are meant to lead to increased opportunities for inclusion in society, that is, career counselling is aimed at shielding individuals from social and economic exclusion in society. The study shares accounts of what we consider a discriminative and restrictive approach to counselling influenced by the unconscious biases towards people with disabilities. The active discouragement to avoid specialised careers based on untested assumptions is detrimental and reinforces stereotypical perceptions of what people with disabilities can achieve, especially where the social mobility of many youths with disabilities is intimately linked to their educational backgrounds. This discouragement in effect limits the ability to become self-determined and act with autonomy (Bandura 1989, 1997). Various scholars have argued for what we have found that career aspirations must be nurtured rather than suppressed to address the exclusion and discrimination experienced by people with disabilities and their acceptance as citizens in society (Bam & Ronnie 2020; Barnes 2012; Finkelstein 2007; Lorenzo et al. 2019; 2004). While discouragement may result in achievement or outcome gaps, the study’s findings present another outcome that of resistance. The researchers have not explored the issues of power, control and micro-terrors with depth, but the findings of this study highlight how participants’ individual experiences are representative of a symbolic violence showered in paternalistic advice and mentorship as described by Ratle et al. (2020), another indication of the omission of care. The debate regarding special schools versus mainstreaming continues, and while the merits may be disputed on a case-by-case basis, it is evident through our study that innovation in teaching can be a key differentiator for the experience of a quality education. Considering the South African context, historically advantaged and disadvantaged schools and infrastructure must be considered when considering the career choices and trajectory of students. While legislative advances have been made, inclusion remains a pipe dream. Special schools in South Africa irrespective of the historical divides remain under resourced, and mainstreaming is almost an impossibility for most youth with disabilities representing a major challenge across all systems (Bronfrenbrenner 1979). The acceptance of disability and the internalisation of the experience are not unique to this study (Chen 2015; Santuzzi et al. 2014), but what we have found is that the earlier this experience occurs for students, a self-reported increase in confidence was expressed in transitioning from special schools. In this study, the role of parents, guardians and teachers (Hirano et al. 2018; Miller et al. 2018) and access to information are critical in shaping career discussions and need to be aligned to ensure the best possible transition. Where there is a dearth of information (Gibson & Martin 2019), scholars are disadvantaged.

### Recommendations and implications

The study underscores the urgent need for reshaping education by promoting the integration of people with disabilities into mainstream schools. This shift dismantles barriers and challenges the medicalised view of disability. Governments and education departments must therefore prioritise comprehensive training programmes for teachers and guidance counsellors to raise awareness of unconscious biases and equip educators with practical strategies to foster diverse career aspirations. Furthermore, the authors stress the importance of supporting special schools through improved resource allocation to enhance the quality of education and career guidance they offer. Early intervention, including career exploration and counselling involving parents, guardians and teachers, is essential in bolstering students’ confidence and broadening their career horizons. Lastly, ensuring access to comprehensive career information for students with disabilities is imperative, empowering informed decision-making.

The authors provide a few implications of this study, firstly, the quality of career guidance profoundly impacts the long-term social and economic inclusion of youth with disabilities. Inadequate guidance perpetuates disadvantages, while supportive counselling opens doors to enhanced opportunities. Secondly, the authors highlight the prevalence of unconscious biases among educators and counsellors that limit students’ career choices. Addressing these biases is crucial for equitable opportunities. Additionally, special schools play a pivotal role and need adequate support where inclusive education and innovative teaching can also offer more positive experiences and broader career options. Lastly, early intervention and support are vital for positive outcomes during transitions.
